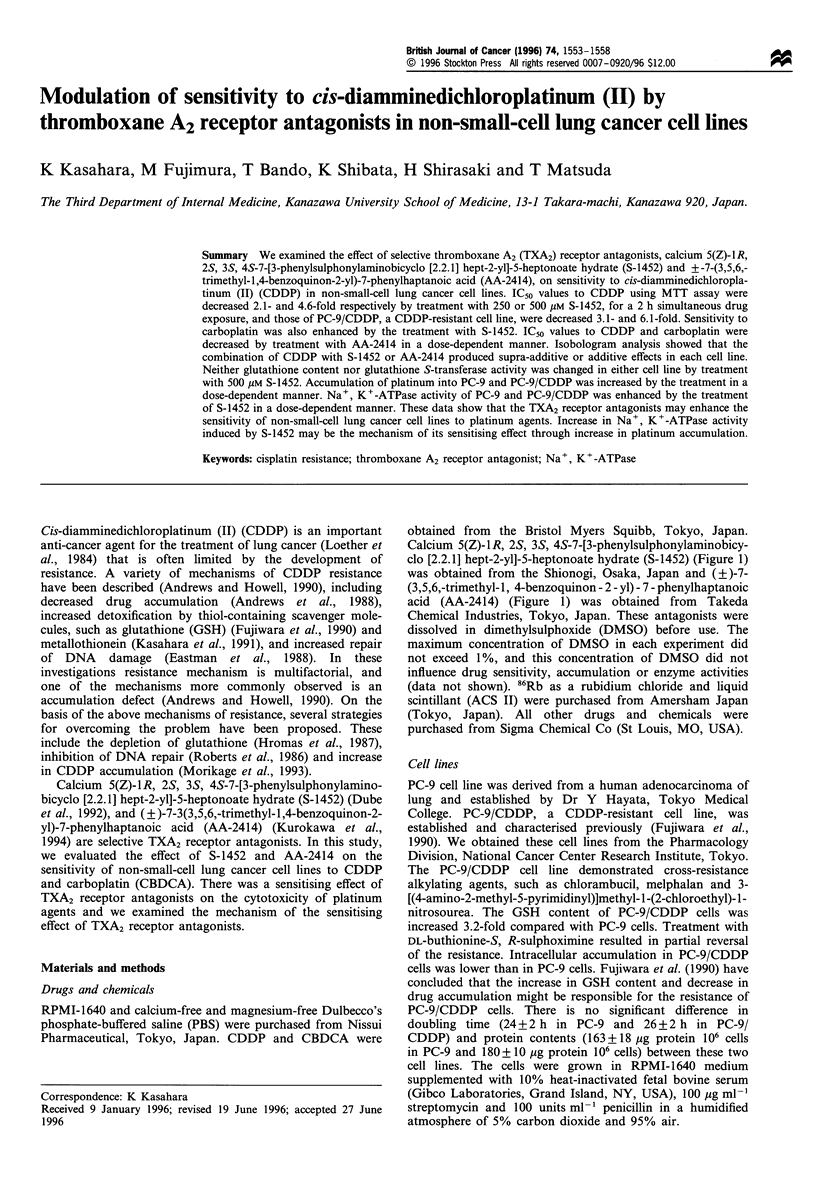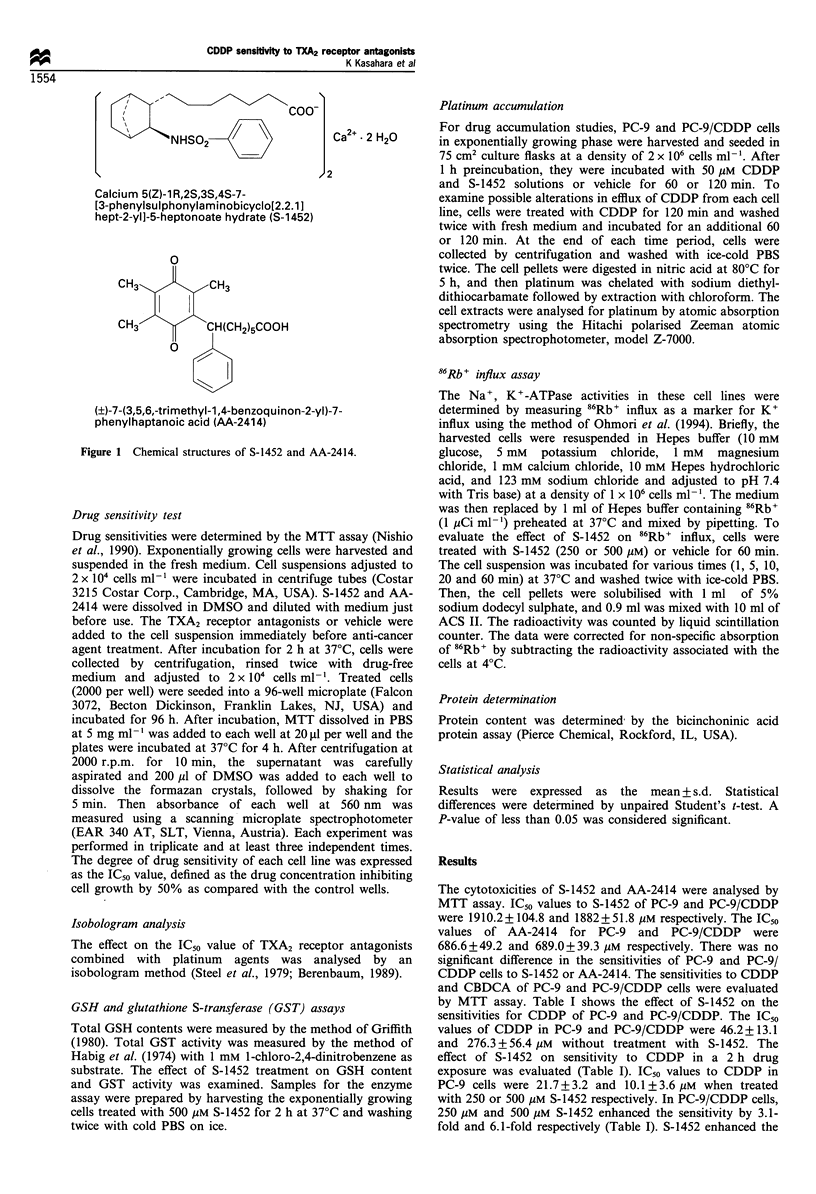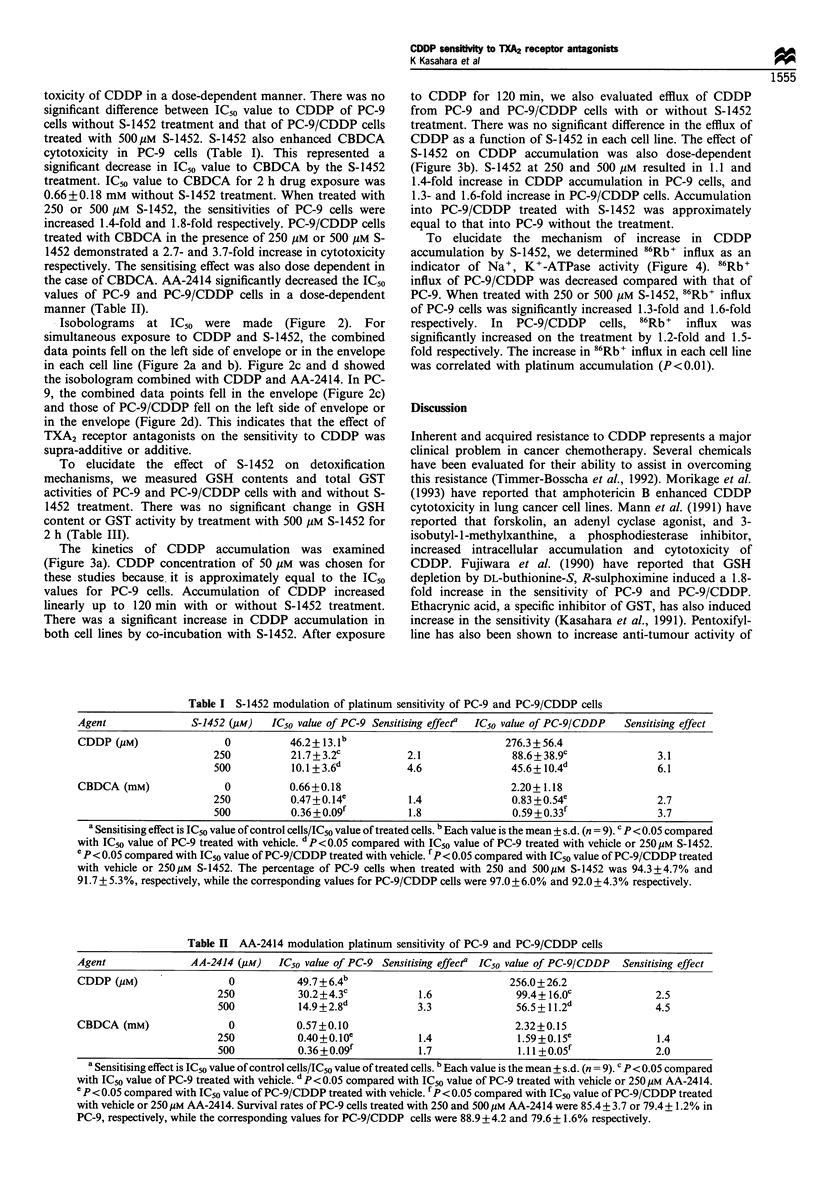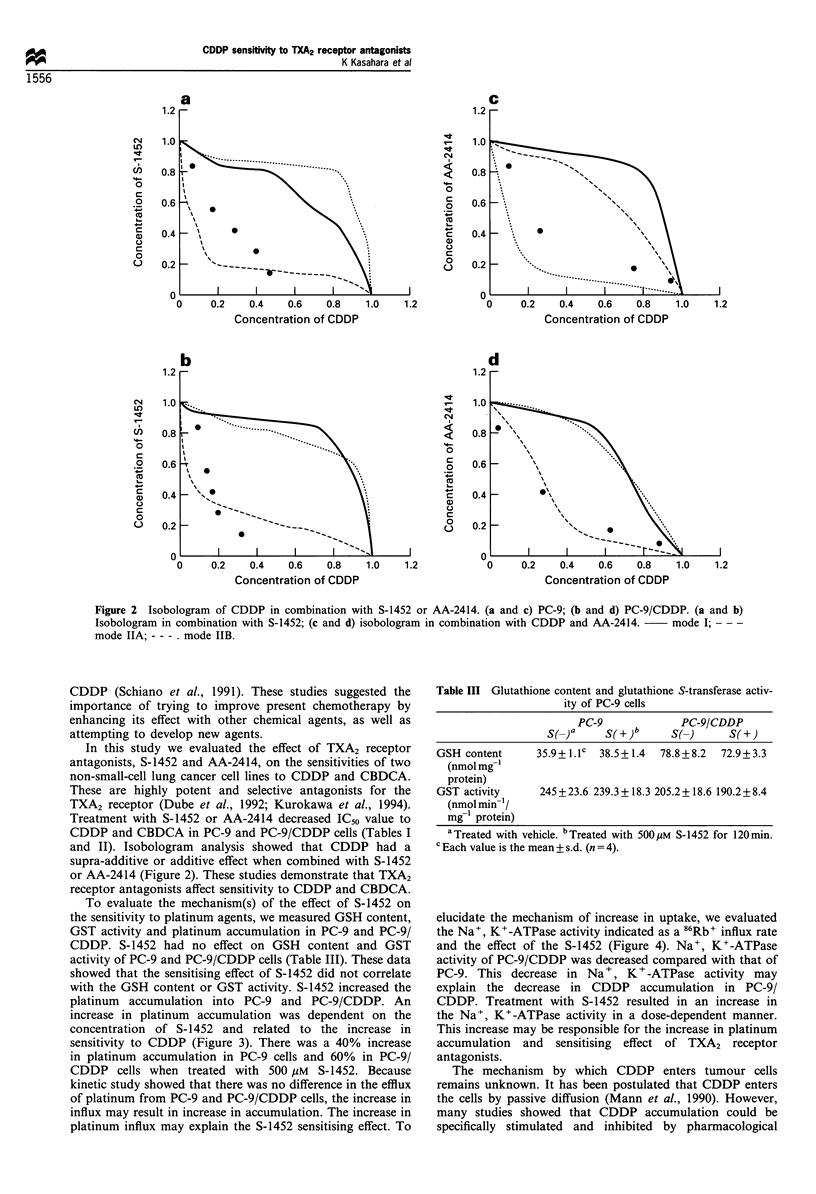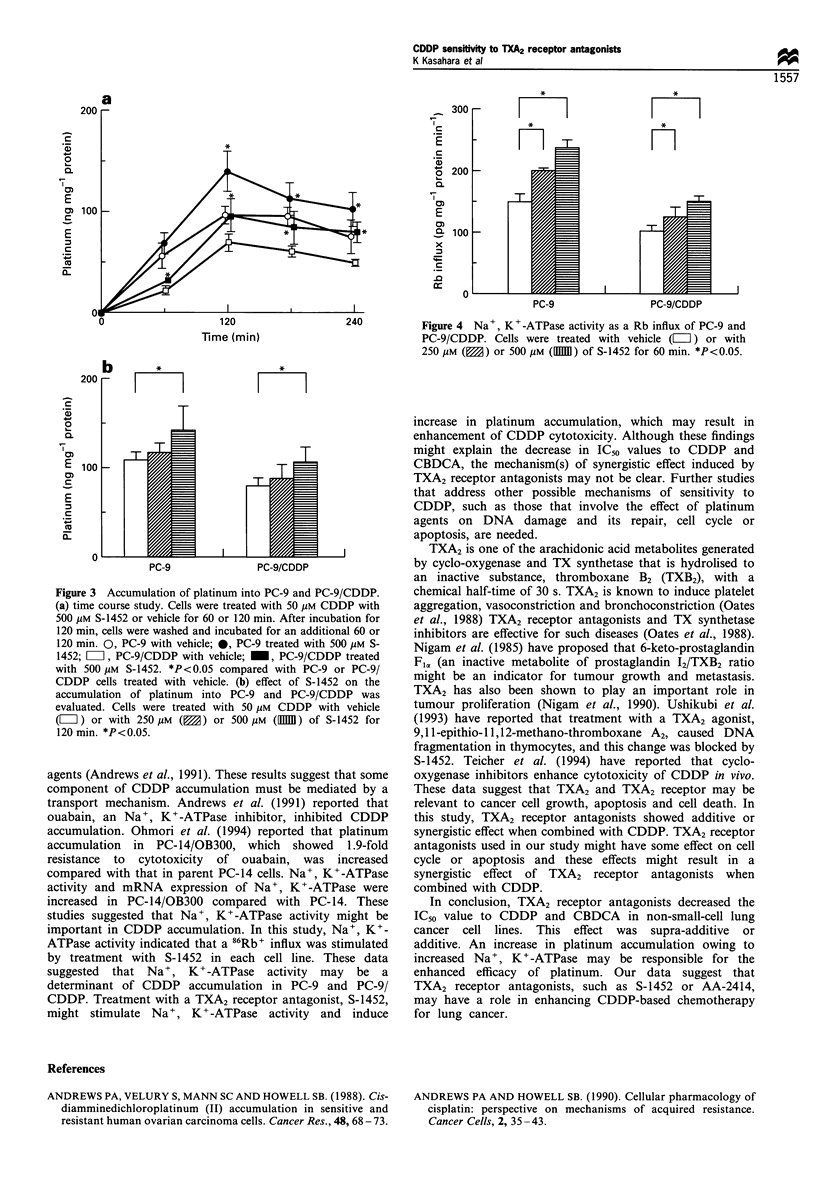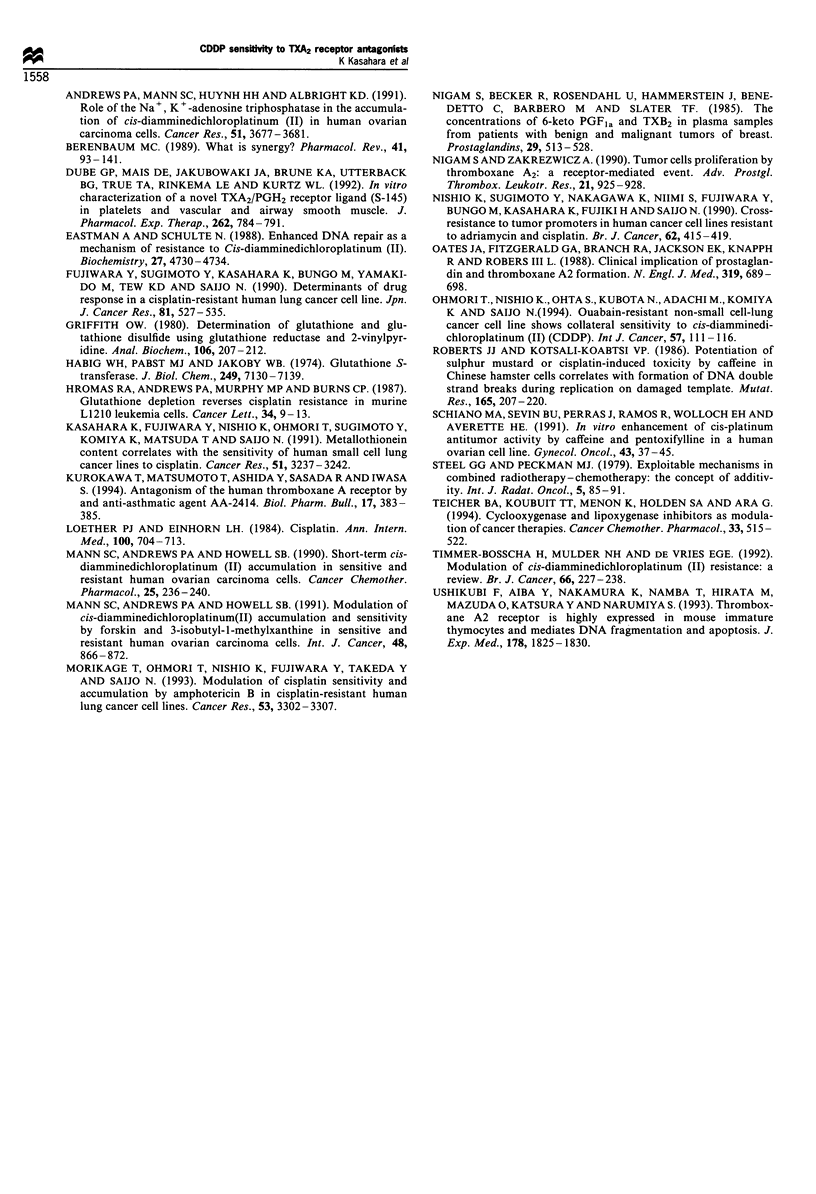# Modulation of sensitivity to cis-diamminedichloroplatinum (II) by thromboxane A2 receptor antagonists in non-small-cell lung cancer cell lines.

**DOI:** 10.1038/bjc.1996.588

**Published:** 1996-11

**Authors:** K. Kasahara, M. Fujimura, T. Bando, K. Shibata, H. Shirasaki, T. Matsuda

**Affiliations:** The Third Department of Internal Medicine, Kanazawa University School of Medicine, Japan.

## Abstract

We examined the effect of selective thromboxane A2 (TXA2) receptor antagonists, calcium 5(Z)-1R, 2S, 3S, 4S-7-[3-phenylsulphonylaminobicyclo [2.2.1] hept-2-yl]-5-heptonoate hydrate (S-1452) and +/- -7-(3,5,6,-trimethyl-1,4-benzoquinon-2-yl)-7-phenylhaptanoic acid (AA-2414), on sensitivity to cis-diamminedichloroplatinum (II) (CDDP) in non-small-cell lung cancer cell lines. IC50 values to CDDP using MTT assay were decreased 2.1- and 4.6-fold respectively by treatment with 250 or 500 microM S-1452, for a 2 h simultaneous drug exposure, and those of PC-9/CDDP, a CDDP-resistant cell line, were decreased 3.1- and 6.1-fold. Sensitivity to carboplatin was also enhanced by the treatment with S-1452. IC50 values to CDDP and carboplatin were decreased by treatment with AA-2414 in a dose-dependent manner. Isobologram analysis showed that the combination of CDDP with S-1452 or AA-2414 produced supra-additive or additive effects in each cell line. Neither glutathione content nor glutathione S-transferase activity was changed in either cell line by treatment with 500 microM S-1452. Accumulation of platinum into PC-9 and PC-9/CDDP was increased by the treatment in a dose-dependent manner. Na+, K+-ATPase activity of PC-9 and PC-9/CDDP was enhanced by the treatment of S-1452 in a dose-dependent manner. These data show that the TXA2 receptor antagonists may enhance the sensitivity of non-small-cell lung cancer cell lines to platinum agents. Increase in Na+, K+-ATPase activity induced by S-1452 may be the mechanism of its sensitising effect through increase in platinum accumulation.


British Journal of Cancer (1996) 74, 1553-1558
© 1996 Stockton Press All rights reserved 0007-0920/96 $12.00

### Modulation of sensitivity to *cis*-diamminedichloroplatinum (II) by thromboxane $A_2$ receptor antagonists in non-small-cell lung cancer cell lines

K Kasahara, M Fujimura, T Bando, K Shibata, H Shirasaki and T Matsuda

*The Third Department of Internal Medicine, Kanazawa University School of Medicine, 13-1 Takara-machi, Kanazawa 920, Japan.*

**Summary** We examined the effect of selective thromboxane $A_2$ ($TXA_2$) receptor antagonists, calcium 5(Z)-1*R*, 2*S*, 3*S*, 4*S*-7-[3-phenylsulphonylaminobicyclo [2.2.1] hept-2-yl]-5-heptonoate hydrate (S-1452) and ±-7-(3,5,6,-trimethyl-1,4-benzoquinon-2-yl)-7-phenylhaptanoic acid (AA-2414), on sensitivity to *cis*-diamminedichloroplatinum (II) (CDDP) in non-small-cell lung cancer cell lines. $IC_{50}$ values to CDDP using MTT assay were decreased 2.1- and 4.6-fold respectively by treatment with 250 or 500 $\mu$M S-1452, for a 2 h simultaneous drug exposure, and those of PC-9/CDDP, a CDDP-resistant cell line, were decreased 3.1- and 6.1-fold. Sensitivity to carboplatin was also enhanced by the treatment with S-1452. $IC_{50}$ values to CDDP and carboplatin were decreased by treatment with AA-2414 in a dose-dependent manner. Isobologram analysis showed that the combination of CDDP with S-1452 or AA-2414 produced supra-additive or additive effects in each cell line. Neither glutathione content nor glutathione *S*-transferase activity was changed in either cell line by treatment with 500 $\mu$M S-1452. Accumulation of platinum into PC-9 and PC-9/CDDP was increased by the treatment in a dose-dependent manner. $Na^+$, $K^+$-ATPase activity of PC-9 and PC-9/CDDP was enhanced by the treatment of S-1452 in a dose-dependent manner. These data show that the $TXA_2$ receptor antagonists may enhance the sensitivity of non-small-cell lung cancer cell lines to platinum agents. Increase in $Na^+$, $K^+$-ATPase activity induced by S-1452 may be the mechanism of its sensitising effect through increase in platinum accumulation.

**Keywords:** cisplatin resistance; thromboxane $A_2$ receptor antagonist; $Na^+$, $K^+$-ATPase

*Cis*-diamminedichloroplatinum (II) (CDDP) is an important anti-cancer agent for the treatment of lung cancer (Loether *et al.*, 1984) that is often limited by the development of resistance. A variety of mechanisms of CDDP resistance have been described (Andrews and Howell, 1990), including decreased drug accumulation (Andrews *et al.*, 1988), increased detoxification by thiol-containing scavenger molecules, such as glutathione (GSH) (Fujiwara *et al.*, 1990) and metallothionein (Kasahara *et al.*, 1991), and increased repair of DNA damage (Eastman *et al.*, 1988). In these investigations resistance mechanism is multifactorial, and one of the mechanisms more commonly observed is an accumulation defect (Andrews and Howell, 1990). On the basis of the above mechanisms of resistance, several strategies for overcoming the problem have been proposed. These include the depletion of glutathione (Hromas *et al.*, 1987), inhibition of DNA repair (Roberts *et al.*, 1986) and increase in CDDP accumulation (Morikage *et al.*, 1993).

Calcium 5(Z)-1*R*, 2*S*, 3*S*, 4*S*-7-[3-phenylsulphonylamino-bicyclo [2.2.1] hept-2-yl]-5-heptonoate hydrate (S-1452) (Dube *et al.*, 1992), and (±)-7-3(3,5,6,-trimethyl-1,4-benzoquinon-2-yl)-7-phenylhaptanoic acid (AA-2414) (Kurokawa *et al.*, 1994) are selective $TXA_2$ receptor antagonists. In this study, we evaluated the effect of S-1452 and AA-2414 on the sensitivity of non-small-cell lung cancer cell lines to CDDP and carboplatin (CBDCA). There was a sensitising effect of $TXA_2$ receptor antagonists on the cytotoxicity of platinum agents and we examined the mechanism of the sensitising effect of $TXA_2$ receptor antagonists.

### Materials and methods

### Drugs and chemicals

RPMI-1640 and calcium-free and magnesium-free Dulbecco's phosphate-buffered saline (PBS) were purchased from Nissui Pharmaceutical, Tokyo, Japan. CDDP and CBDCA were obtained from the Bristol Myers Squibb, Tokyo, Japan. Calcium 5(Z)-1*R*, 2*S*, 3*S*, 4*S*-7-[3-phenylsulphonylaminobicyclo [2.2.1] hept-2-yl]-5-heptonoate hydrate (S-1452) (Figure 1) was obtained from the Shionogi, Osaka, Japan and (±)-7-(3,5,6,-trimethyl-1, 4-benzoquinon-2-yl)-7-phenylhaptanoic acid (AA-2414) (Figure 1) was obtained from Takeda Chemical Industries, Tokyo, Japan. These antagonists were dissolved in dimethylsulphoxide (DMSO) before use. The maximum concentration of DMSO in each experiment did not exceed 1%, and this concentration of DMSO did not influence drug sensitivity, accumulation or enzyme activities (data not shown). $^{86}$Rb as a rubidium chloride and liquid scintillant (ACS II) were purchased from Amersham Japan (Tokyo, Japan). All other drugs and chemicals were purchased from Sigma Chemical Co (St Louis, MO, USA).

### Cell lines

PC-9 cell line was derived from a human adenocarcinoma of lung and established by Dr Y Hayata, Tokyo Medical College. PC-9/CDDP, a CDDP-resistant cell line, was established and characterised previously (Fujiwara *et al.*, 1990). We obtained these cell lines from the Pharmacology Division, National Cancer Center Research Institute, Tokyo. The PC-9/CDDP cell line demonstrated cross-resistance alkylating agents, such as chlorambucil, melphalan and 3-[(4-amino-2-methyl-5-pyrimidinyl)]methyl-1-(2-chloroethyl)-1-nitrosourea. The GSH content of PC-9/CDDP cells was increased 3.2-fold compared with PC-9 cells. Treatment with DL-buthionine-*S*, *R*-sulphoximine resulted in partial reversal of the resistance. Intracellular accumulation in PC-9/CDDP cells was lower than in PC-9 cells. Fujiwara *et al.* (1990) have concluded that the increase in GSH content and decrease in drug accumulation might be responsible for the resistance of PC-9/CDDP cells. There is no significant difference in doubling time (24±2 h in PC-9 and 26±2 h in PC-9/CDDP) and protein contents (163±18 $\mu$g protein $10^6$ cells in PC-9 and 180±10 $\mu$g protein $10^6$ cells) between these two cell lines. The cells were grown in RPMI-1640 medium supplemented with 10% heat-inactivated fetal bovine serum (Gibco Laboratories, Grand Island, NY, USA), 100 $\mu$g ml$^{-1}$ streptomycin and 100 units ml$^{-1}$ penicillin in a humidified atmosphere of 5% carbon dioxide and 95% air.

Correspondence: K Kasahara
Received 9 January 1996; revised 19 June 1996; accepted 27 June 1996

Calcium 5(Z)-1R,2S,3S,4S-7-
[3-phenylsulphonylaminobicyclo[2.2.1]
hept-2-yl]-5-heptonoate hydrate (S-1452)

(±)-7-(3,5,6,-trimethyl-1,4-benzoquinon-2-yl)-7-
phenylhaptanoic acid (AA-2414)

**Figure 1** Chemical structures of S-1452 and AA-2414.

*Drug sensitivity test*

Drug sensitivities were determined by the MTT assay (Nishio *et al.*, 1990). Exponentially growing cells were harvested and suspended in the fresh medium. Cell suspensions adjusted to $2\times10^4$ cells $ml^{-1}$ were incubated in centrifuge tubes (Costar 3215 Costar Corp., Cambridge, MA, USA). S-1452 and AA-2414 were dissolved in DMSO and diluted with medium just before use. The $TXA_2$ receptor antagonists or vehicle were added to the cell suspension immediately before anti-cancer agent treatment. After incubation for 2 h at 37°C, cells were collected by centrifugation, rinsed twice with drug-free medium and adjusted to $2\times10^4$ cells $ml^{-1}$. Treated cells (2000 per well) were seeded into a 96-well microplate (Falcon 3072, Becton Dickinson, Franklin Lakes, NJ, USA) and incubated for 96 h. After incubation, MTT dissolved in PBS at 5 mg $ml^{-1}$ was added to each well at 20 µl per well and the plates were incubated at 37°C for 4 h. After centrifugation at 2000 r.p.m. for 10 min, the supernatant was carefully aspirated and 200 µl of DMSO was added to each well to dissolve the formazan crystals, followed by shaking for 5 min. Then absorbance of each well at 560 nm was measured using a scanning microplate spectrophotometer (EAR 340 AT, SLT, Vienna, Austria). Each experiment was performed in triplicate and at least three independent times. The degree of drug sensitivity of each cell line was expressed as the $IC_{50}$ value, defined as the drug concentration inhibiting cell growth by 50% as compared with the control wells.

*Isobologram analysis*

The effect on the $IC_{50}$ value of $TXA_2$ receptor antagonists combined with platinum agents was analysed by an isobologram method (Steel *et al.*, 1979; Berenbaum, 1989).

*GSH and glutathione S-transferase (GST) assays*

Total GSH contents were measured by the method of Griffith (1980). Total GST activity was measured by the method of Habig *et al.* (1974) with 1 mM 1-chloro-2,4-dinitrobenzene as substrate. The effect of S-1452 treatment on GSH content and GST activity was examined. Samples for the enzyme assay were prepared by harvesting the exponentially growing cells treated with 500 µM S-1452 for 2 h at 37°C and washing twice with cold PBS on ice.

*Platinum accumulation*

For drug accumulation studies, PC-9 and PC-9/CDDP cells in exponentially growing phase were harvested and seeded in 75 $cm^2$ culture flasks at a density of $2\times10^6$ cells $ml^{-1}$. After 1 h preincubation, they were incubated with 50 µM CDDP and S-1452 solutions or vehicle for 60 or 120 min. To examine possible alterations in efflux of CDDP from each cell line, cells were treated with CDDP for 120 min and washed twice with fresh medium and incubated for an additional 60 or 120 min. At the end of each time period, cells were collected by centrifugation and washed with ice-cold PBS twice. The cell pellets were digested in nitric acid at 80°C for 5 h, and then platinum was chelated with sodium diethyldithiocarbamate followed by extraction with chloroform. The cell extracts were analysed for platinum by atomic absorption spectrometry using the Hitachi polarised Zeeman atomic absorption spectrophotometer, model Z-7000.

*$^{86}Rb^+$ influx assay*

The $Na^+$, $K^+$-ATPase activities in these cell lines were determined by measuring $^{86}Rb^+$ influx as a marker for $K^+$ influx using the method of Ohmori *et al.* (1994). Briefly, the harvested cells were resuspended in Hepes buffer (10 mM glucose, 5 mM potassium chloride, 1 mM magnesium chloride, 1 mM calcium chloride, 10 mM Hepes hydrochloric acid, and 123 mM sodium chloride and adjusted to pH 7.4 with Tris base) at a density of $1\times10^6$ cells $ml^{-1}$. The medium was then replaced by 1 ml of Hepes buffer containing $^{86}Rb^+$ (1 µCi $ml^{-1}$) preheated at 37°C and mixed by pipetting. To evaluate the effect of S-1452 on $^{86}Rb^+$ influx, cells were treated with S-1452 (250 or 500 µM) or vehicle for 60 min. The cell suspension was incubated for various times (1, 5, 10, 20 and 60 min) at 37°C and washed twice with ice-cold PBS. Then, the cell pellets were solubilised with 1 ml of 5% sodium dodecyl sulphate, and 0.9 ml was mixed with 10 ml of ACS II. The radioactivity was counted by liquid scintillation counter. The data were corrected for non-specific absorption of $^{86}Rb^+$ by subtracting the radioactivity associated with the cells at 4°C.

*Protein determination*

Protein content was determined by the bicinchoninic acid protein assay (Pierce Chemical, Rockford, IL, USA).

*Statistical analysis*

Results were expressed as the mean ± s.d. Statistical differences were determined by unpaired Student's *t*-test. A *P*-value of less than 0.05 was considered significant.

### Results

The cytotoxicities of S-1452 and AA-2414 were analysed by MTT assay. $IC_{50}$ values to S-1452 of PC-9 and PC-9/CDDP were 1910.2 ± 104.8 and 1882 ± 51.8 µM respectively. The $IC_{50}$ values of AA-2414 for PC-9 and PC-9/CDDP were 686.6 ± 49.2 and 689.0 ± 39.3 µM respectively. There was no significant difference in the sensitivities of PC-9 and PC-9/CDDP cells to S-1452 or AA-2414. The sensitivities to CDDP and CBDCA of PC-9 and PC-9/CDDP cells were evaluated by MTT assay. Table I shows the effect of S-1452 on the sensitivities for CDDP of PC-9 and PC-9/CDDP. The $IC_{50}$ values of CDDP in PC-9 and PC-9/CDDP were 46.2 ± 13.1 and 276.3 ± 56.4 µM without treatment with S-1452. The effect of S-1452 on sensitivity to CDDP in a 2 h drug exposure was evaluated (Table I). $IC_{50}$ values to CDDP in PC-9 cells were 21.7 ± 3.2 and 10.1 ± 3.6 µM when treated with 250 or 500 µM S-1452 respectively. In PC-9/CDDP cells, 250 µM and 500 µM S-1452 enhanced the sensitivity by 3.1-fold and 6.1-fold respectively (Table I). S-1452 enhanced the toxicity of CDDP in a dose-dependent manner. There was no significant difference between $IC_{50}$ value to CDDP of PC-9 cells without S-1452 treatment and that of PC-9/CDDP cells treated with 500 μM S-1452. S-1452 also enhanced CBDCA cytotoxicity in PC-9 cells (Table I). This represented a significant decrease in $IC_{50}$ value to CBDCA by the S-1452 treatment. $IC_{50}$ value to CBDCA for 2 h drug exposure was 0.66±0.18 mM without S-1452 treatment. When treated with 250 or 500 μM S-1452, the sensitivities of PC-9 cells were increased 1.4-fold and 1.8-fold respectively. PC-9/CDDP cells treated with CBDCA in the presence of 250 μM or 500 μM S-1452 demonstrated a 2.7- and 3.7-fold increase in cytotoxicity respectively. The sensitising effect was also dose dependent in the case of CBDCA. AA-2414 significantly decreased the $IC_{50}$ values of PC-9 and PC-9/CDDP cells in a dose-dependent manner (Table II).

Isobolograms at $IC_{50}$ were made (Figure 2). For simultaneous exposure to CDDP and S-1452, the combined data points fell on the left side of envelope or in the envelope in each cell line (Figure 2a and b). Figure 2c and d showed the isobologram combined with CDDP and AA-2414. In PC-9, the combined data points fell in the envelope (Figure 2c) and those of PC-9/CDDP fell on the left side of envelope or in the envelope (Figure 2d). This indicates that the effect of $TXA_2$ receptor antagonists on the sensitivity to CDDP was supra-additive or additive.

To elucidate the effect of S-1452 on detoxification mechanisms, we measured GSH contents and total GST activities of PC-9 and PC-9/CDDP cells with and without S-1452 treatment. There was no significant change in GSH content or GST activity by treatment with 500 μM S-1452 for 2 h (Table III).

The kinetics of CDDP accumulation was examined (Figure 3a). CDDP concentration of 50 μM was chosen for these studies because it is approximately equal to the $IC_{50}$ values for PC-9 cells. Accumulation of CDDP increased linearly up to 120 min with or without S-1452 treatment. There was a significant increase in CDDP accumulation in both cell lines by co-incubation with S-1452. After exposure to CDDP for 120 min, we also evaluated efflux of CDDP from PC-9 and PC-9/CDDP cells with or without S-1452 treatment. There was no significant difference in the efflux of CDDP as a function of S-1452 in each cell line. The effect of S-1452 on CDDP accumulation was also dose-dependent (Figure 3b). S-1452 at 250 and 500 μM resulted in 1.1 and 1.4-fold increase in CDDP accumulation in PC-9 cells, and 1.3- and 1.6-fold increase in PC-9/CDDP cells. Accumulation into PC-9/CDDP treated with S-1452 was approximately equal to that into PC-9 without the treatment.

To elucidate the mechanism of increase in CDDP accumulation by S-1452, we determined $^{86}Rb^+$ influx as an indicator of $Na^+$, $K^+$-ATPase activity (Figure 4). $^{86}Rb^+$ influx of PC-9/CDDP was decreased compared with that of PC-9. When treated with 250 or 500 μM S-1452, $^{86}Rb^+$ influx of PC-9 cells was significantly increased 1.3-fold and 1.6-fold respectively. In PC-9/CDDP cells, $^{86}Rb^+$ influx was significantly increased on the treatment by 1.2-fold and 1.5-fold respectively. The increase in $^{86}Rb^+$ influx in each cell line was correlated with platinum accumulation ($P<0.01$).

### Discussion

Inherent and acquired resistance to CDDP represents a major clinical problem in cancer chemotherapy. Several chemicals have been evaluated for their ability to assist in overcoming this resistance (Timmer-Bosscha *et al*., 1992). Morikage *et al.* (1993) have reported that amphotericin B enhanced CDDP cytotoxicity in lung cancer cell lines. Mann *et al.* (1991) have reported that forskolin, an adenyl cyclase agonist, and 3-isobutyl-1-methylxanthine, a phosphodiesterase inhibitor, increased intracellular accumulation and cytotoxicity of CDDP. Fujiwara *et al.* (1990) have reported that GSH depletion by DL-buthionine-*S*, *R*-sulphoximine induced a 1.8-fold increase in the sensitivity of PC-9 and PC-9/CDDP. Ethacrynic acid, a specific inhibitor of GST, has also induced increase in the sensitivity (Kasahara *et al*., 1991). Pentoxifylline has also been shown to increase anti-tumour activity of

**Table I** S-1452 modulation of platinum sensitivity of PC-9 and PC-9/CDDP cells

| *Agent* | *S-1452 (μM)* | *$IC_{50}$ value of PC-9* | *Sensitising effect[a]* | *$IC_{50}$ value of PC-9/CDDP* | *Sensitising effect* |
|---|---|---|---|---|---|
| CDDP (μM) | 0 | 46.2±13.1[b] | | 276.3±56.4 | |
| | 250 | 21.7±3.2[c] | 2.1 | 88.6±38.9[c] | 3.1 |
| | 500 | 10.1±3.6[d] | 4.6 | 45.6±10.4[d] | 6.1 |
| CBDCA (mM) | 0 | 0.66±0.18 | | 2.20±1.18 | |
| | 250 | 0.47±0.14[e] | 1.4 | 0.83±0.54[e] | 2.7 |
| | 500 | 0.36±0.09[f] | 1.8 | 0.59±0.33[f] | 3.7 |

[a] Sensitising effect is $IC_{50}$ value of control cells/$IC_{50}$ value of treated cells. [b] Each value is the mean±s.d. ($n=9$). [c] $P<0.05$ compared with $IC_{50}$ value of PC-9 treated with vehicle. [d] $P<0.05$ compared with $IC_{50}$ value of PC-9 treated with vehicle or 250 μM S-1452. [e] $P<0.05$ compared with $IC_{50}$ value of PC-9/CDDP treated with vehicle. [f] $P<0.05$ compared with $IC_{50}$ value of PC-9/CDDP treated with vehicle or 250 μM S-1452. The percentage of PC-9 cells when treated with 250 and 500 μM S-1452 was 94.3±4.7% and 91.7±5.3%, respectively, while the corresponding values for PC-9/CDDP cells were 97.0±6.0% and 92.0±4.3% respectively.

**Table II** AA-2414 modulation platinum sensitivity of PC-9 and PC-9/CDDP cells

| *Agent* | *AA-2414 (μM)* | *$IC_{50}$ value of PC-9* | *Sensitising effect[a]* | *$IC_{50}$ value of PC-9/CDDP* | *Sensitising effect* |
|---|---|---|---|---|---|
| CDDP (μM) | 0 | 49.7±6.4[b] | | 256.0±26.2 | |
| | 250 | 30.2±4.3[c] | 1.6 | 99.4±16.0[c] | 2.5 |
| | 500 | 14.9±2.8[d] | 3.3 | 56.5±11.2[d] | 4.5 |
| CBDCA (mM) | 0 | 0.57±0.10 | | 2.32±0.15 | |
| | 250 | 0.40±0.10[e] | 1.4 | 1.59±0.15[e] | 1.4 |
| | 500 | 0.36±0.09[f] | 1.7 | 1.11±0.05[f] | 2.0 |

[a] Sensitising effect is $IC_{50}$ value of control cells/$IC_{50}$ value of treated cells. [b] Each value is the mean±s.d. ($n=9$). [c] $P<0.05$ compared with $IC_{50}$ value of PC-9 treated with vehicle. [d] $P<0.05$ compared with $IC_{50}$ value of PC-9 treated with vehicle or 250 μM AA-2414. [e] $P<0.05$ compared with $IC_{50}$ value of PC-9/CDDP treated with vehicle. [f] $P<0.05$ compared with $IC_{50}$ value of PC-9/CDDP treated with vehicle or 250 μM AA-2414. Survival rates of PC-9 cells treated with 250 and 500 μM AA-2414 were 85.4±3.7 or 79.4±1.2% in PC-9, respectively, while the corresponding values for PC-9/CDDP cells were 88.9±4.2 and 79.6±1.6% respectively.

**Figure 2** Isobologram of CDDP in combination with S-1452 or AA-2414. (**a** and **c**) PC-9; (**b** and **d**) PC-9/CDDP. (**a** and **b**) Isobologram in combination with S-1452; (**c** and **d**) isobologram in combination with CDDP and AA-2414. —— mode I; – – – mode IIA; - - - - . mode IIB.

CDDP (Schiano *et al.*, 1991). These studies suggested the importance of trying to improve present chemotherapy by enhancing its effect with other chemical agents, as well as attempting to develop new agents.

In this study we evaluated the effect of $TXA_2$ receptor antagonists, S-1452 and AA-2414, on the sensitivities of two non-small-cell lung cancer cell lines to CDDP and CBDCA. These are highly potent and selective antagonists for the $TXA_2$ receptor (Dube *et al.*, 1992; Kurokawa *et al.*, 1994). Treatment with S-1452 or AA-2414 decreased $IC_{50}$ value to CDDP and CBDCA in PC-9 and PC-9/CDDP cells (Tables I and II). Isobologram analysis showed that CDDP had a supra-additive or additive effect when combined with S-1452 or AA-2414 (Figure 2). These studies demonstrate that $TXA_2$ receptor antagonists affect sensitivity to CDDP and CBDCA.

To evaluate the mechanism(s) of the effect of S-1452 on the sensitivity to platinum agents, we measured GSH content, GST activity and platinum accumulation in PC-9 and PC-9/CDDP. S-1452 had no effect on GSH content and GST activity of PC-9 and PC-9/CDDP cells (Table III). These data showed that the sensitising effect of S-1452 did not correlate with the GSH content or GST activity. S-1452 increased the platinum accumulation into PC-9 and PC-9/CDDP. An increase in platinum accumulation was dependent on the concentration of S-1452 and related to the increase in sensitivity to CDDP (Figure 3). There was a 40% increase in platinum accumulation in PC-9 cells and 60% in PC-9/CDDP cells when treated with 500 $\mu$M S-1452. Because kinetic study showed that there was no difference in the efflux of platinum from PC-9 and PC-9/CDDP cells, the increase in influx may result in increase in accumulation. The increase in platinum influx may explain the S-1452 sensitising effect. To

**Table III** Glutathione content and glutathione *S*-transferase activity of PC-9 cells

| | *PC-9* | | *PC-9/CDDP* | |
|---|---|---|---|---|
| | *S(−)*[a] | *S(+)*[b] | *S(−)* | *S(+)* |
| GSH content (nmol $mg^{-1}$ protein) | 35.9±1.1[c] | 38.5±1.4 | 78.8±8.2 | 72.9±3.3 |
| GST activity (nmol $min^{-1}$/ $mg^{-1}$ protein) | 245±23.6 | 239.3±18.3 | 205.2±18.6 | 190.2±8.4 |

[a]Treated with vehicle. [b]Treated with 500 $\mu$M S-1452 for 120 min. [c]Each value is the mean±s.d. ($n$=4).

elucidate the mechanism of increase in uptake, we evaluated the $Na^+$, $K^+$-ATPase activity indicated as a $^{86}Rb^+$ influx rate and the effect of the S-1452 (Figure 4). $Na^+$, $K^+$-ATPase activity of PC-9/CDDP was decreased compared with that of PC-9. This decrease in $Na^+$, $K^+$-ATPase activity may explain the decrease in CDDP accumulation in PC-9/CDDP. Treatment with S-1452 resulted in an increase in the $Na^+$, $K^+$-ATPase activity in a dose-dependent manner. This increase may be responsible for the increase in platinum accumulation and sensitising effect of $TXA_2$ receptor antagonists.

The mechanism by which CDDP enters tumour cells remains unknown. It has been postulated that CDDP enters the cells by passive diffusion (Mann *et al.*, 1990). However, many studies showed that CDDP accumulation could be specifically stimulated and inhibited by pharmacological

**Figure 3** Accumulation of platinum into PC-9 and PC-9/CDDP. (**a**) time course study. Cells were treated with 50 $\mu$M CDDP with 500 $\mu$M S-1452 or vehicle for 60 or 120 min. After incubation for 120 min, cells were washed and incubated for an additional 60 or 120 min. ○, PC-9 with vehicle; ●, PC-9 treated with 500 $\mu$M S-1452; □, PC-9/CDDP with vehicle; ■, PC-9/CDDP treated with 500 $\mu$M S-1452. *$P<0.05$ compared with PC-9 or PC-9/CDDP cells treated with vehicle. (**b**) effect of S-1452 on the accumulation of platinum into PC-9 and PC-9/CDDP was evaluated. Cells were treated with 50 $\mu$M CDDP with vehicle (□) or with 250 $\mu$M (▨) or 500 $\mu$M (▥) of S-1452 for 120 min. *$P<0.05$.

**Figure 4** $Na^+$, $K^+$-ATPase activity as a Rb influx of PC-9 and PC-9/CDDP. Cells were treated with vehicle (□) or with 250 $\mu$M (▨) or 500 $\mu$M (▥) of S-1452 for 60 min. *$P<0.05$.

agents (Andrews *et al.*, 1991). These results suggest that some component of CDDP accumulation must be mediated by a transport mechanism. Andrews *et al.* (1991) reported that ouabain, an $Na^+$, $K^+$-ATPase inhibitor, inhibited CDDP accumulation. Ohmori *et al.* (1994) reported that platinum accumulation in PC-14/OB300, which showed 1.9-fold resistance to cytotoxicity of ouabain, was increased compared with that in parent PC-14 cells. $Na^+$, $K^+$-ATPase activity and mRNA expression of $Na^+$, $K^+$-ATPase were increased in PC-14/OB300 compared with PC-14. These studies suggested that $Na^+$, $K^+$-ATPase activity might be important in CDDP accumulation. In this study, $Na^+$, $K^+$-ATPase activity indicated that a $^{86}Rb^+$ influx was stimulated by treatment with S-1452 in each cell line. These data suggested that $Na^+$, $K^+$-ATPase activity may be a determinant of CDDP accumulation in PC-9 and PC-9/CDDP. Treatment with a $TXA_2$ receptor antagonist, S-1452, might stimulate $Na^+$, $K^+$-ATPase activity and induce increase in platinum accumulation, which may result in enhancement of CDDP cytotoxicity. Although these findings might explain the decrease in $IC_{50}$ values to CDDP and CBDCA, the mechanism(s) of synergistic effect induced by $TXA_2$ receptor antagonists may not be clear. Further studies that address other possible mechanisms of sensitivity to CDDP, such as those that involve the effect of platinum agents on DNA damage and its repair, cell cycle or apoptosis, are needed.

$TXA_2$ is one of the arachidonic acid metabolites generated by cyclo-oxygenase and TX synthetase that is hydrolised to an inactive substance, thromboxane $B_2$ ($TXB_2$), with a chemical half-time of 30 s. $TXA_2$ is known to induce platelet aggregation, vasoconstriction and bronchoconstriction (Oates *et al.*, 1988) $TXA_2$ receptor antagonists and TX synthetase inhibitors are effective for such diseases (Oates *et al.*, 1988). Nigam *et al.* (1985) have proposed that 6-keto-prostaglandin $F_{1\alpha}$ (an inactive metabolite of prostaglandin $I_2/TXB_2$ ratio might be an indicator for tumour growth and metastasis. $TXA_2$ has also been shown to play an important role in tumour proliferation (Nigam *et al.*, 1990). Ushikubi *et al.* (1993) have reported that treatment with a $TXA_2$ agonist, 9,11-epithio-11,12-methano-thromboxane $A_2$, caused DNA fragmentation in thymocytes, and this change was blocked by S-1452. Teicher *et al.* (1994) have reported that cyclo-oxygenase inhibitors enhance cytotoxicity of CDDP *in vivo*. These data suggest that $TXA_2$ and $TXA_2$ receptor may be relevant to cancer cell growth, apoptosis and cell death. In this study, $TXA_2$ receptor antagonists showed additive or synergistic effect when combined with CDDP. $TXA_2$ receptor antagonists used in our study might have some effect on cell cycle or apoptosis and these effects might result in a synergistic effect of $TXA_2$ receptor antagonists when combined with CDDP.

In conclusion, $TXA_2$ receptor antagonists decreased the $IC_{50}$ value to CDDP and CBDCA in non-small-cell lung cancer cell lines. This effect was supra-additive or additive. An increase in platinum accumulation owing to increased $Na^+$, $K^+$-ATPase may be responsible for the enhanced efficacy of platinum. Our data suggest that $TXA_2$ receptor antagonists, such as S-1452 or AA-2414, may have a role in enhancing CDDP-based chemotherapy for lung cancer.

### References

ANDREWS PA, VELURY S, MANN SC AND HOWELL SB. (1988). *Cis*-diamminedichloroplatinum (II) accumulation in sensitive and resistant human ovarian carcinoma cells. *Cancer Res.*, **48,** 68–73.

ANDREWS PA AND HOWELL SB. (1990). Cellular pharmacology of cisplatin: perspective on mechanisms of acquired resistance. *Cancer Cells*, **2,** 35–43.

ANDREWS PA, MANN SC, HUYNH HH AND ALBRIGHT KD. (1991). Role of the $Na^+$, $K^+$-adenosine triphosphatase in the accumulation of *cis*-diamminedichloroplatinum (II) in human ovarian carcinoma cells. *Cancer Res.*, **51**, 3677–3681.

BERENBAUM MC. (1989). What is synergy? *Pharmacol. Rev.*, **41**, 93–141.

DUBE GP, MAIS DE, JAKUBOWAKI JA, BRUNE KA, UTTERBACK BG, TRUE TA, RINKEMA LE AND KURTZ WL. (1992). *In vitro* characterization of a novel $TXA_2/PGH_2$ receptor ligand (S-145) in platelets and vascular and airway smooth muscle. *J. Pharmacol. Exp. Therap.*, **262**, 784–791.

EASTMAN A AND SCHULTE N. (1988). Enhanced DNA repair as a mechanism of resistance to *Cis*-diamminedichloroplatinum (II). *Biochemistry*, **27**, 4730–4734.

FUJIWARA Y, SUGIMOTO Y, KASAHARA K, BUNGO M, YAMAKIDO M, TEW KD AND SAIJO N. (1990). Determinants of drug response in a cisplatin-resistant human lung cancer cell line. *Jpn. J. Cancer Res.*, **81**, 527–535.

GRIFFITH OW. (1980). Determination of glutathione and glutathione disulfide using glutathione reductase and 2-vinylpyridine. *Anal. Biochem.*, **106**, 207–212.

HABIG WH, PABST MJ AND JAKOBY WB. (1974). Glutathione *S*-transferase. *J. Biol. Chem.*, **249**, 7130–7139.

HROMAS RA, ANDREWS PA, MURPHY MP AND BURNS CP. (1987). Glutathione depletion reverses cisplatin resistance in murine L1210 leukemia cells. *Cancer Lett.*, **34**, 9–13.

KASAHARA K, FUJIWARA Y, NISHIO K, OHMORI T, SUGIMOTO Y, KOMIYA K, MATSUDA T AND SAIJO N. (1991). Metallothionein content correlates with the sensitivity of human small cell lung cancer lines to cisplatin. *Cancer Res.*, **51**, 3237–3242.

KUROKAWA T, MATSUMOTO T, ASHIDA Y, SASADA R AND IWASA S. (1994). Antagonism of the human thromboxane A receptor by and anti-asthmatic agent AA-2414. *Biol. Pharm. Bull.*, **17**, 383–385.

LOETHER PJ AND EINHORN LH. (1984). Cisplatin. *Ann. Intern. Med.*, **100**, 704–713.

MANN SC, ANDREWS PA AND HOWELL SB. (1990). Short-term *cis*-diamminedichloroplatinum (II) accumulation in sensitive and resistant human ovarian carcinoma cells. *Cancer Chemother. Pharmacol.*, **25**, 236–240.

MANN SC, ANDREWS PA AND HOWELL SB. (1991). Modulation of *cis*-diamminedichloroplatinum(II) accumulation and sensitivity by forskin and 3-isobutyl-1-methylxanthine in sensitive and resistant human ovarian carcinoma cells. *Int. J. Cancer*, **48**, 866–872.

MORIKAGE T, OHMORI T, NISHIO K, FUJIWARA Y, TAKEDA Y AND SAIJO N. (1993). Modulation of cisplatin sensitivity and accumulation by amphotericin B in cisplatin-resistant human lung cancer cell lines. *Cancer Res.*, **53**, 3302–3307.

NIGAM S, BECKER R, ROSENDAHL U, HAMMERSTEIN J, BENEDETTO C, BARBERO M AND SLATER TF. (1985). The concentrations of 6-keto $PGF_{1a}$ and $TXB_2$ in plasma samples from patients with benign and malignant tumors of breast. *Prostaglandins*, **29**, 513–528.

NIGAM S AND ZAKREZWICZ A. (1990). Tumor cells proliferation by thromboxane $A_2$: a receptor-mediated event. *Adv. Prostgl. Thrombox. Leukotr. Res.*, **21**, 925–928.

NISHIO K, SUGIMOTO Y, NAKAGAWA K, NIIMI S, FUJIWARA Y, BUNGO M, KASAHARA K, FUJIKI H AND SAIJO N. (1990). Cross-resistance to tumor promoters in human cancer cell lines resistant to adriamycin and cisplatin. *Br. J. Cancer*, **62**, 415–419.

OATES JA, FITZGERALD GA, BRANCH RA, JACKSON EK, KNAPPH R AND ROBERS III L. (1988). Clinical implication of prostaglandin and thromboxane A2 formation. *N. Engl. J. Med.*, **319**, 689–698.

OHMORI T., NISHIO K., OHTA S., KUBOTA N., ADACHI M., KOMIYA K AND SAIJO N.(1994). Ouabain-resistant non-small cell-lung cancer cell line shows collateral sensitivity to *cis*-diamminedichloroplatinum (II) (CDDP). *Int J. Cancer*, **57**, 111–116.

ROBERTS JJ AND KOTSALI-KOABTSI VP. (1986). Potentiation of sulphur mustard or cisplatin-induced toxicity by caffeine in Chinese hamster cells correlates with formation of DNA double strand breaks during replication on damaged template. *Mutat. Res.*, **165**, 207–220.

SCHIANO MA, SEVIN BU, PERRAS J, RAMOS R, WOLLOCH EH AND AVERETTE HE. (1991). *In vitro* enhancement of cis-platinum antitumor activity by caffeine and pentoxifylline in a human ovarian cell line. *Gynecol. Oncol.*, **43**, 37–45.

STEEL GG AND PECKMAN MJ. (1979). Exploitable mechanisms in combined radiotherapy–chemotherapy: the concept of additivity. *Int. J. Radat. Oncol.*, **5**, 85–91.

TEICHER BA, KOUBUIT TT, MENON K, HOLDEN SA AND ARA G. (1994). Cyclooxygenase and lipoxygenase inhibitors as modulation of cancer therapies. *Cancer Chemother. Pharmacol.*, **33**, 515–522.

TIMMER-BOSSCHA H, MULDER NH AND DE VRIES EGE. (1992). Modulation of *cis*-diamminedichloroplatinum (II) resistance: a review. *Br. J. Cancer*, **66**, 227–238.

USHIKUBI F, AIBA Y, NAKAMURA K, NAMBA T, HIRATA M, MAZUDA O, KATSURA Y AND NARUMIYA S. (1993). Thromboxane A2 receptor is highly expressed in mouse immature thymocytes and mediates DNA fragmentation and apoptosis. *J. Exp. Med.*, **178**, 1825–1830.